# Microscopic evidence of strong interactions between chemical vapor deposited 2D MoS_2_ film and SiO_2_ growth template

**DOI:** 10.1186/s40580-021-00262-x

**Published:** 2021-04-09

**Authors:** Woonbae Sohn, Ki Chang Kwon, Jun Min Suh, Tae Hyung Lee, Kwang Chul Roh, Ho Won Jang

**Affiliations:** 1 Department of Materials Science and Engineering, Research Institute of Advanced Materials, Seoul National University, Seoul, 08826 Republic of Korea; 2 Energy Storage Materials Centre, Korea Institute of Ceramic Engineering and Technology, Jinju, 52851 Republic of Korea; 3 Advanced Institute of Convergence Technology, Seoul National University, Suwon, 16229 Republic of Korea

**Keywords:** MoS_2_, Large-scale growth, Chemical bonding, Electron energy loss spectroscopy

## Abstract

Two-dimensional MoS_2_ film can grow on oxide substrates including Al_2_O_3_ and SiO_2_. However, it cannot grow usually on non-oxide substrates such as a bare Si wafer using chemical vapor deposition. To address this issue, we prepared as-synthesized and transferred MoS_2_ (AS-MoS_2_ and TR-MoS_2_) films on SiO_2_/Si substrates and studied the effect of the SiO_2_ layer on the atomic and electronic structure of the MoS_2_ films using spherical aberration-corrected scanning transition electron microscopy (STEM) and electron energy loss spectroscopy (EELS). The interlayer distance between MoS_2_ layers film showed a change at the AS-MoS_2_/SiO_2_ interface, which is attributed to the formation of S–O chemical bonding at the interface, whereas the TR-MoS_2_/SiO_2_ interface showed only van der Waals interactions. Through STEM and EELS studies, we confirmed that there exists a bonding state in addition to the van der Waals force, which is the dominant interaction between MoS_2_ and SiO_2_. The formation of S–O bonding at the AS-MoS_2_/SiO_2_ interface layer suggests that the sulfur atoms at the termination layer in the MoS_2_ films are bonded to the oxygen atoms of the SiO_2_ layer during chemical vapor deposition. Our results indicate that the S–O bonding feature promotes the growth of MoS_2_ thin films on oxide growth templates.

**Supplementary Information:**

The online version contains supplementary material available at 10.1186/s40580-021-00262-x.

## Introduction

Transition metal dichalcogenides, such as MoS_2_, have attracted much interest because of their remarkable electrical, mechanical, thermal, and optical properties. Therefore, they are considered novel materials and suitable for application in optoelectronic devices, water-splitting catalysts, sensors, field-effect transistors, capacitors, and energy storage devices [1–10]. Recent studies have focused on the large-scale growth of MoS_2_ films, which is mainly carried out on Al_2_O_3_ and SiO_2_/Si substrates [11–14]. Through chemical vapor deposition (CVD) and pulsed laser deposition (PLD), MoS_2_ films are well deposited when the Si wafer has a SiO_2_ layer on the top. In contrast, the growth of MoS_2_ fails or MoSi_2_ is synthesized instead of MoS_2_ when CVD growth is carried out on a bare Si wafer even if Si substrate has a native oxide layer on top because the thin native oxide layer can be removed or penetrated during the high temperature synthesis process [4, 15]. Moreover, there are a few studies to show the formation of MoSi_2_ through chemical reactions between MoS_2_ and Si. When the Mo film is sulfurized on a bare Si wafer, the formation of a gas phase SiS_2_ rather than MoS_2_ is presumable. This indicates that making large-scale growth of MoS_2_ directly on Si is hardly achievable. However, when Mo is sulfurized on SiO_2_ substrates, S–O bonding is preferable rather than S–Si bonding, which helps Mo-S bonding followed by growth of a MoS_2_ film. The different tendency of CVD MoS_2_ growth on bare Si and SiO_2_/Si substrates was found in previous studies [4, 15]. To date, it has been reported that some substrates such as SiO_2_/Si and sapphire enable the large-scale deposition of MoS_2_ films. However, microscopic evidence showing the interaction between the chemical-vapor-deposited MoS_2_ and the SiO_2_ growth template has not been observed. Microscopic studies are mainly focused on the atomic and electronic structures of MoS_2_, using transmission electron microscopy (TEM) and aberration-corrected scanning transmission electron microscopy (Cs-corrected STEM), which can directly observe materials on an atomic level. Crystal and atomic structures with various defects have been discovered using a combination of ab-initio calculations [4, 11–14, 16–22].

To investigate the atomic and electronic structure and chemical state of the MoS_2_/SiO_2_ heterostructure, an atomic-scale study using Cs-corrected STEM and EELS with a cross-sectional view should be carried out. The use of Cs-corrected STEM with a probe corrector would enable the direct observation of heterointerfaces at the atomic resolution. Moreover, the changes in the electronic and chemical states would be available in unit-cell resolution. Interfaces between dissimilar compounds provide unusual properties that are attributed to lattice mismatch, strain, chemical bonds, and the formation of secondary interfacial layers [23–27]. The observation of the atomic and electronic structures of MoS_2_/SiO_2_ interfaces not only provides insights on the growth mechanisms and bonding states, but also suggests the possibility of application in various devices. Understanding the nature of the interfaces can provide insights on how the MoS_2_ film is synthesized via CVD and how the MoS_2_ ***`***film on the SiO_2_/Si template can be utilized in electronic devices.

In this work, we investigated the atomic and electronic structures of MoS_2_ (more than 20 layers)/SiO_2_ interfaces on an Si substrate. First, we observed and compared the atomic structure of the AS and TR-MoS_2_ films on the SiO_2_/Si substrates and plotted the (001) plane distance from the interface to the interior of the film. Second, we investigated the chemical bonding state at the AS and TR-MoS_2_/SiO_2_ hetero interfaces. The final aim of this study is figuring out the main reason why MoS_2_ is deposited only on oxide substrates such as SiO_2,_ Al_2_O_3_ and understanding how the SiO_2_ layer affects the structures of MoS_2_ films when Mo is sulfurized through CVD [11–13]. We used SiO_2_ for a growth template mainly because other oxides such as Al_2_O_3_ and SrTiO_3_ do not dissolve in HF, disabling transfer process. For this purpose, we carried out a TEM-based analysis with Cs-corrected high annular dark field (HAADF) imaging, and annular bright field (ABF) STEM imaging and EELS with a combination of ab-initio calculations. To the best of our knowledge, this is the first reported paper investigating the influence of the SiO_2_ template on the growth of MoS_2_ films using Cs-corrected STEM and EELS with a cross-sectional view.

## Experimental details

### MoS_*2*_ film deposition

SiO_2_(300 nm)/Si wafers were cleaned with a standard piranha solution (3:1 mixture of H_2_SO_4_ and H_2_O_2_) using conventional cleaning procedures followed by ultrasonication in acetone, isopropyl alcohol, and deionized (DI) water. To obtain hydrophilic surfaces on the SiO_2_/Si wafers, O_2_ plasma and UV-O_3_ surface treatments were sequentially performed for 15 min. A 10 nm-thick Mo thin film was deposited on a SiO_2_/Si substrate using an E-beam evaporator. (Rocky Mountain Vacuum Tech, Englewood, CO, USA). The base pressure, E-beam voltage, and current were 10^–6^ Torr, 7.3 kV, and 70 mA, respectively, and the deposition rate was approximately 0.1 Å/s. Sulfurization of the Mo thin film was performed by CVD at 900 °C for 30 min. After sulfurization, the thin films were annealed for the crystallization of the MoS_2_ films.

### TEM sample preparation

Due to the weak interaction between the MoS_2_ film and SiO_2_ layer, MoS_2_ films are peeled off from SiO_2_ when cross-section TEM specimen preparation using polishing. To minimize mechanical damage, we carried out FIB TEM sample preparation followed by nanomilling (Fischione 1040). Nanomill is similar to precision ion polishing, except that the milling area can be selected during nanomill process. Thus, nanomill is the optimal method for the preparation of the FIB TEM specimens.

### Raman, XPS, TEM and STEM/EELS characterization

Raman and X-ray photoelectron spectroscopy were carried out to analyze vibration modes and surface chemical states of the MoS_2_ films using LABRAM HR Evolution and AXIS-Hsi. The Mo to S ratio of the MoS_2_ films are calculated by dividing intensity or the area of each elements by factor of Mo or S elements [4].

The TEM analysis was divided into 2 steps. First, we obtained bright-field TEM and high-resolution TEM images to confirm the quality and thickness of the TEM specimen. In this step, we analyzed the MoS_2_ film using JEOL JEM-2100F. In the next step, to investigate the atomic structure of the interfaces, Cs-corrected high-resolution STEM images were obtained with a Cs-probe corrected TEM instrument (JEOL JEM ARM 200F). STEM imaging with a spherical aberration corrector provided clearer images with a spatial resolution of 80 pm. Thus, Cs-corrected HR-STEM imaging enabled the identification of elements as well as the location of atomic positions with high accuracy.

### Theoretical calculation

For the calculations, the MoS_2_-SiO_2_ heterostructure supercell was composed of 8 Si layers of SiO_2_, a monolayer of MoS_2_, and a 15 Å vacuum layer to prevent the interaction between layers. Both sides of the SiO_2_ slab were reconstructed surfaces, which have a lower surface energy compared to a pristine surface according to a previous study [29]. Before constructing the heterostructure, the unit cells of SiO_2_ and MoS_2_ were first relaxed; the lattice parameters were a = b = 4.896 Å for SiO_2_ and a = b = 3.161 Å for MoS_2_. Subsequently, along the x–y plane, SiO_2_ in 2 × 2 lateral periodicity and MoS_2_ in 3 × 3 lateral periodicity was stacked together. Considering that the monolayer MoS_2_ is much more prone to deformation than bulk SiO_2_, the x–y plane lattice parameter of 2 × 2 SiO_2_ was employed as that of a heterostructure supercell. This allowed the MoS_2_ layer to expand along the x and y directions with a lattice mismatch of approximately 3.4%. In addition, the position of the SiO_2_ layer was fixed during the relaxation of the heterostructure for the same reason mentioned above. All structural relaxations and free energy calculations were performed with the *Vienna *ab initio simulation package (VASP) [31, 32] based on density functional theory (DFT) [33, 34]. For the replacement of the core electrons, a projector augmented wave (PAW) [35, 36] scheme was implemented. The exchange–correlation energy was described through the generalized gradient approximation (GGA) using the Perdew-Burke-Emzerhof (PBE) functional [37]. The kinetic cutoff for the plane-wave basis was 400 eV. The Brillouin zone for 3 × 3 × 1 k-point sampling was constructed with a gamma-centered grid. For electronic self-consistency and force tolerance, criteria of 5–10 eV and 0.01 eV/A were applied.

## Results and discussion

### The growth of the MoS_2_ film and imaging of the MoS_2_/SiO_2_ interface

**Fig. 1:**
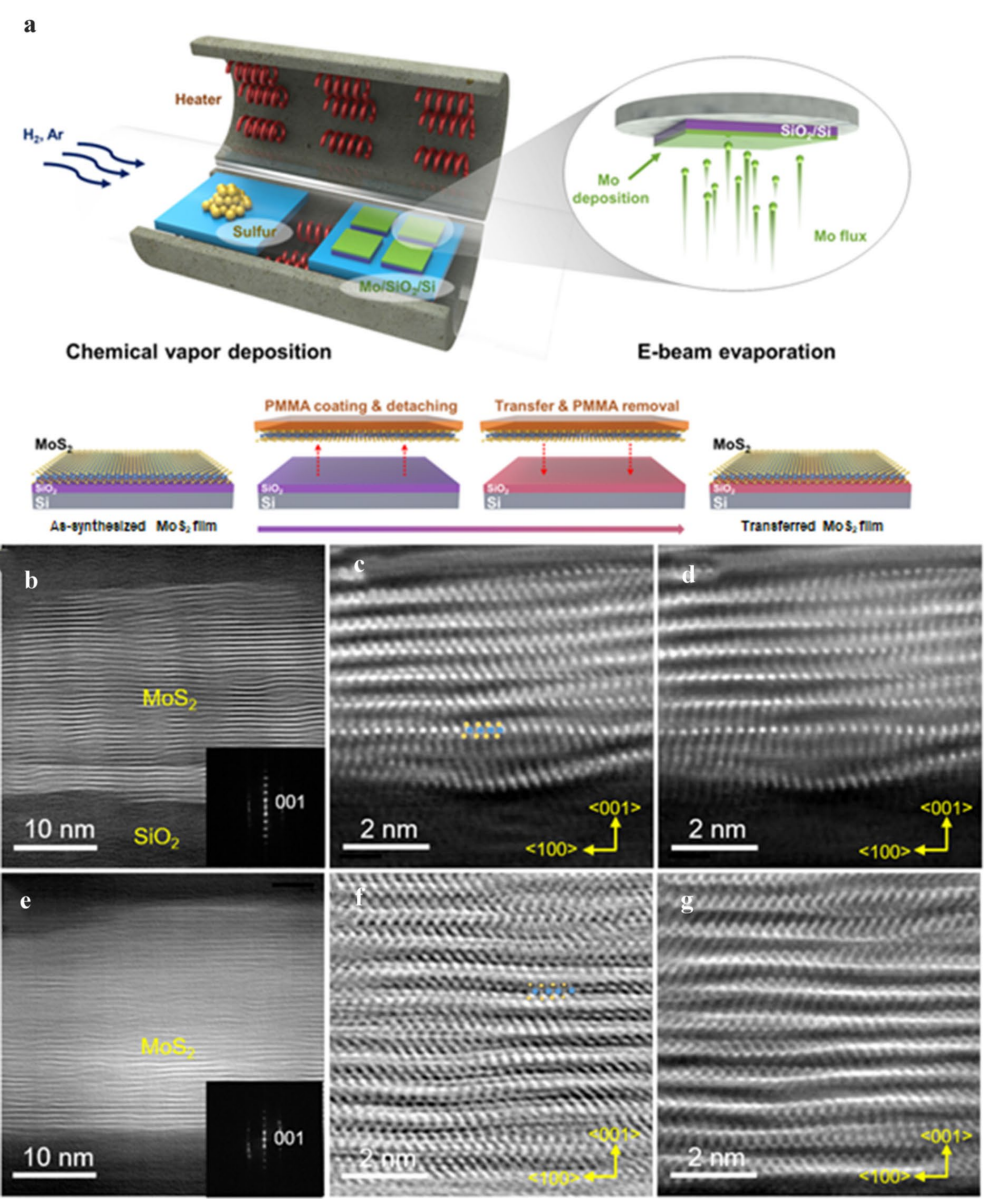
**a** Schematic illustration of the MoS_2_ film deposition and transfer. STEM images of MoS_2_ film on SiO_2_/Si. **b** Low-magnification HAADF STEM, **c** Cs-corrected ABF STEM, and **d** Cs-corrected HAADF STEM images of AS-MoS_2_ film. **e** Low-magnification HAADF STEM, **f** Cs-corrected ABF STEM, and **g** Cs- corrected HAADF STEM images of TR-MoS_2_ film

The growth and transfer process of the MoS_2_ film is shown in Fig. 1(a). The experimental details are explained in the Sect. 2.1. The growth of the AS-MoS_2_ film was demonstrated using Raman spectroscopy, as shown in Additional file 1: Figure S1. The Raman spectrum showed two characteristic Raman vibration modes, E^1^_2g_ and A_1g_, which indicated that the MoS_2_ film grew laterally on the Si/SiO_2_ substrate.

Due to the weak interaction between the TMD film and the substrate, which hinders the observation of the structure of the interfaces when the TEM sample is prepared using mechanical polishing, we prepared a TEM specimen using a focused ion beam (FIB) followed by nanomill using a Fischione 1040 nanomill. Additional file 1: Figure S2 depicts the result using the prepared AS and TR-MoS_2_ films, which are in line with the Raman spectrum. Figure 1(b) and (c) is the HAADF STEM image and shows that the MoS_2_ film was laterally aligned on the amorphous silicon oxide layer. Since the SiO_2_ growth template is not atomically smooth, the AS-MoS_2_/SiO_2_ interface seems to be rough. Figure 1(c) is a contrast-inverted ABF STEM image. As ABF STEM imaging is efficient in detecting light elements, especially sulfur, the atomic configuration in Fig. 1(d) shows clearer image contrast than that in Fig. 1(c). The TR-MoS_2_ film also showed comparatively well laterally aligned MoS_2_ sheets at the surface and in the interior of the layer as shown in Fig. 1(e–g). In addition, the TR-MoS_2_ film showed no significant difference in the overall lattice structure and interlayer distance.

### Interlayer distance of the AS and TR-MoS_2_ films depending on number of layers

**Fig. 2:**
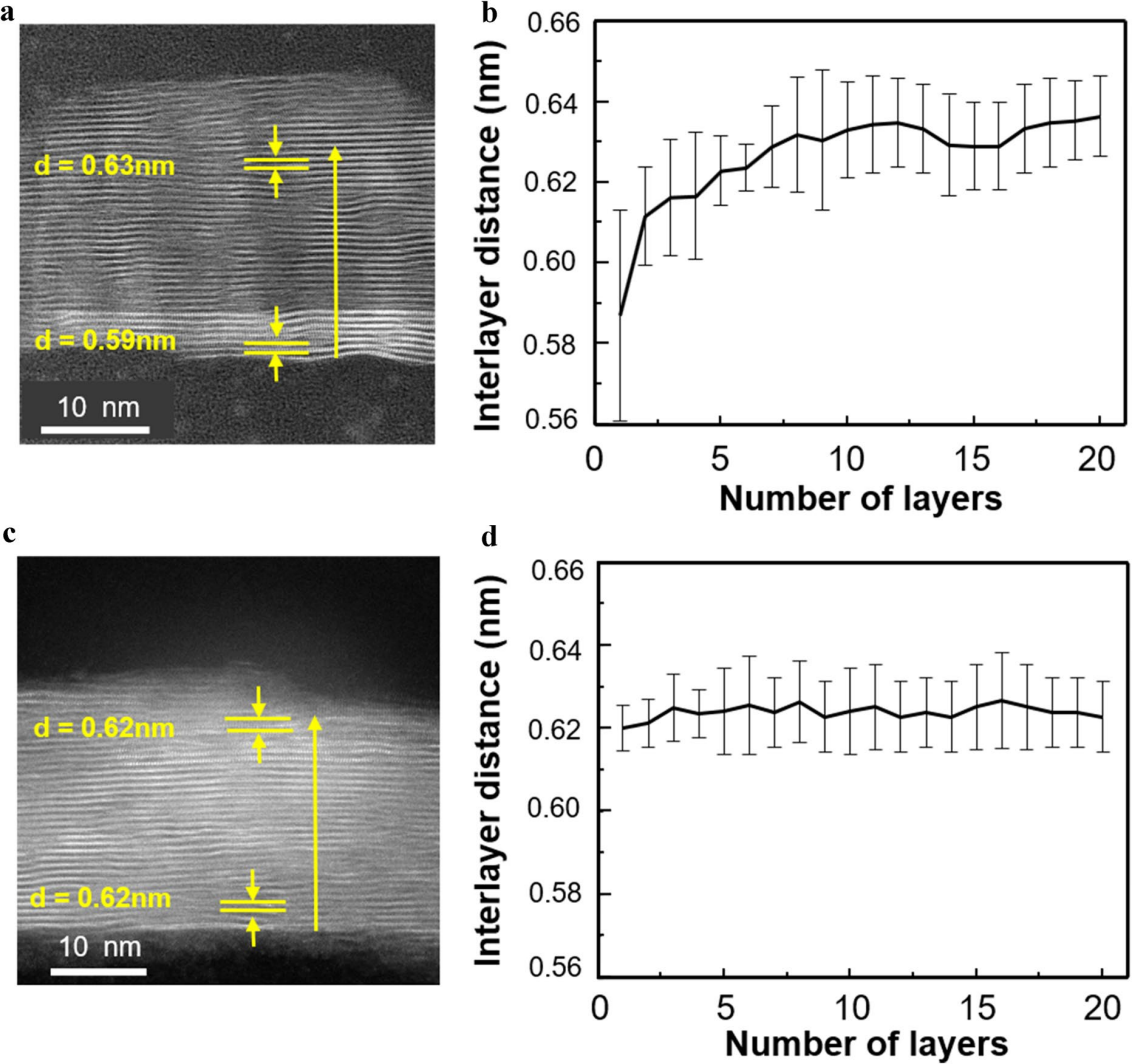
Changes in the interlayer distance at the MoS_2_/SiO_2_ interface. **a** Position at which the interlayer distance values were measured in the AS-MoS_2_ films. **b** Plot of the interlayer distance of AS-MoS_2_. The black solid line represents the average value of the interlayer distance with error. **c** Position at which the interlayer distance values were measured in the TR-MoS_2_ films. **d** Plot of the interlayer distance of TR-MoS_2_. The black solid line represents the average value of the distance with error

To quantify the effect of the amorphous SiO_2_ layer on the atomic structure of the MoS_2_ film, we plotted the changes in the out-of-plane distance at 16 different positions and calculated the average interlayer distance with error bars. Each measured distance of the AS and TR-MoS_2_ films is depicted in Additional file 1: Figure S3. As shown in Fig. 2(a) and (b), at the AS-MoS_2_/SiO_2_ interface, the interlayer distance was up to 3.7% shorter than that of the AS-MoS_2_ film, whereas there was no significant change in the interlayer distance in the TR-MoS_2_ film, as shown in Fig. 2(c) and (d). The huge error bar is not originated from the roughness of the of the SiO_2_ substrate, but irregular S–O bond at the MoS_2_/SiO_2_ interface, making the shape of the MoS_2_ surface wavy. Despite of this roughness, the difference in tendency of interlayer distance of AS and TR-MoS_2_ films is non-negligible. This suggests that when MoS_2_ films are grown on SiO_2_ via the sulfurization of the Mo film, SiO_2_ not only plays a significant role as a growth template but also influences the atomic structure of the AS-MoS_2_ film at the MoS_2_/SiO_2_ interface. Considering that only van der Waals interactions occur between the MoS_2_ layers in the bulk, the change in interlayer distance is attributed to the chemical bonding between AS-MoS_2_ and SiO_2_.

**Fig. 3:**
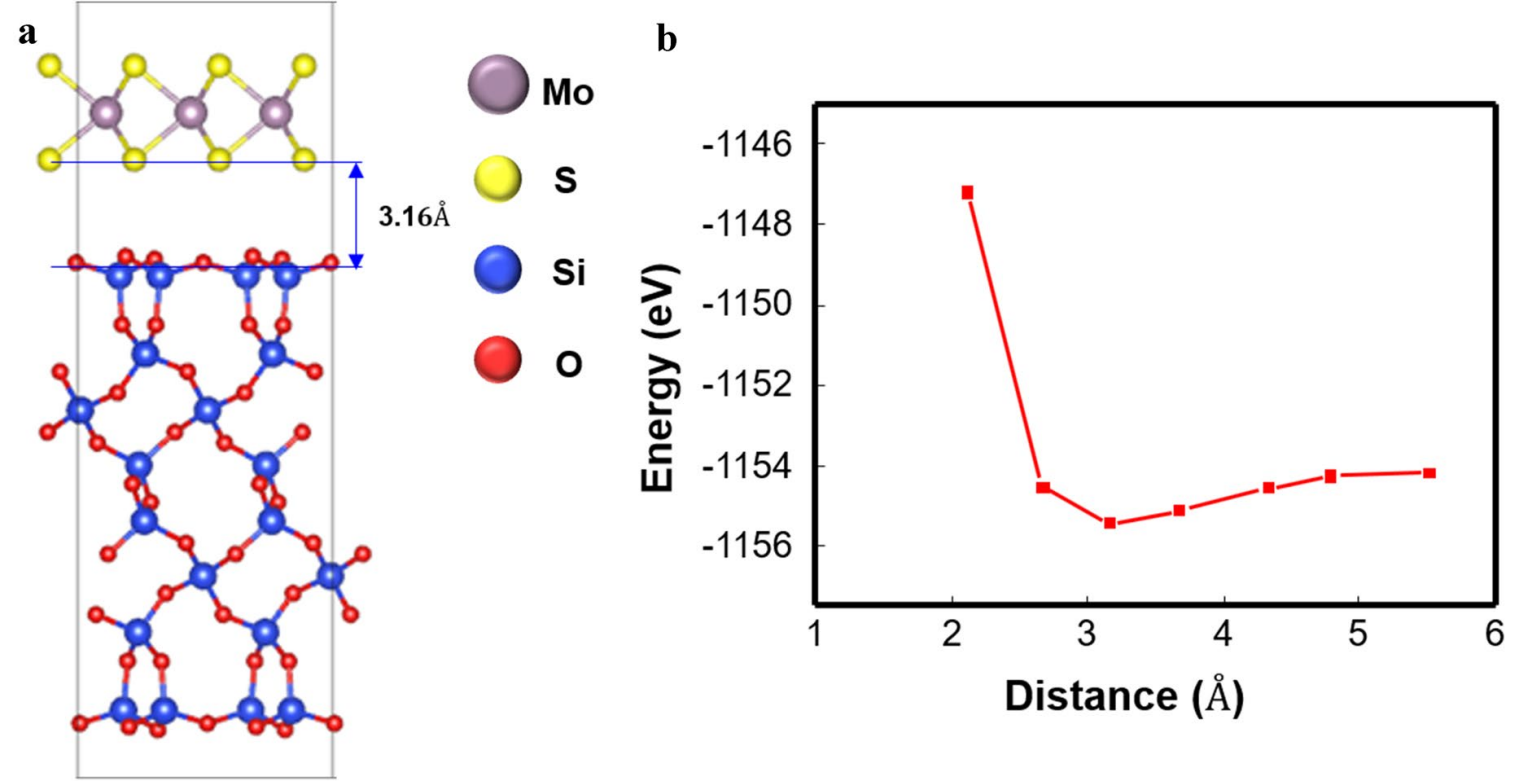
**a** Atomistic model of the MoS_2_ layer on the single-crystal SiO_2_. **b** Formation energy as a function of SiO_2_-MoS_2_ distance

Next, we carried out ab-initio calculations to suggest an atomic model based on STEM images. We compared the equivalent distance between SiO_2_ (single crystal)-MoS_2_ and MoS_2_-MoS_2_ with the assumption that the MoS_2_/SiO_2_ interface is S–O-terminated. As shown in Fig. 3(a), the shortest distance between SiO_2_-MoS_2_ was calculated to be 3.16 Å, which was slightly longer than the MoS_2_-MoS_2_ distance, 3.04 Å. Figure 3(b) illustrates the formation energy as a function of the distance between SiO_2_ and MoS_2_. Figure 3(b) shows that 3.16 Å provides the most stable formation energy in our calculation. Since the calculation assumed that SiO_2_ was a single crystal and considered only the van der Waals interaction, the result of the calculation deviated from that of the experiment. This discrepancy suggests that there is a strong chemical interaction other than the van der Waals force between the AS MoS_2_ film and the SiO_2_ template. This is in contrast with the previous DFT study, which argued that the structure of MoS_2_ is not affected by SiO_2_ [28, 29].

### Chemical bonding at the MoS_2_/SiO_2_ interface

**Fig. 4:**
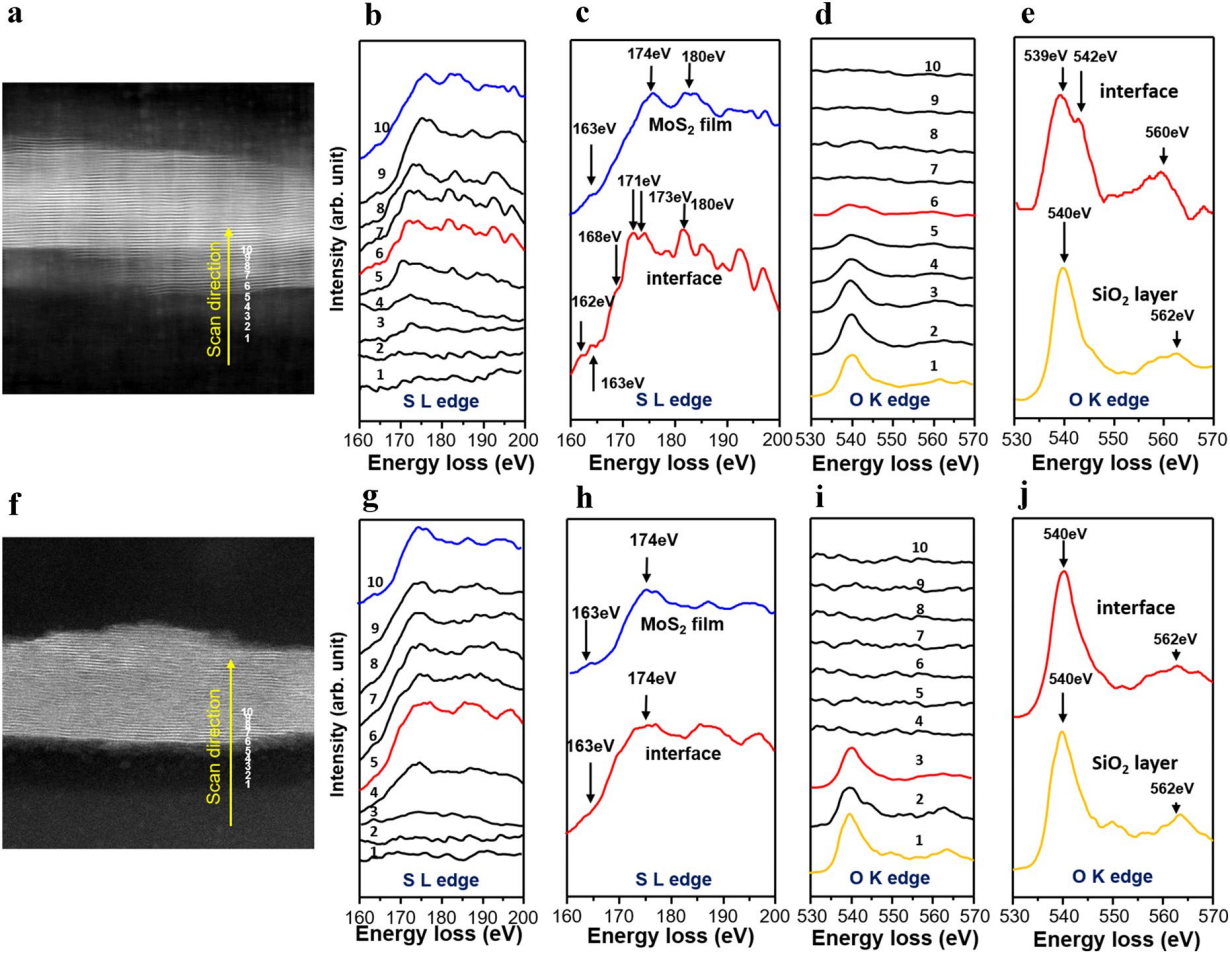
**a** Region of AS-MoS_2_/SiO_2_ in which EELS spectra were obtained. **b** S L-edge of the AS-MoS_2_ film. **c** Comparison between the S L-edges of the AS-MoS_2_ film (blue line) and MoS_2_/SiO_2_ interface (red line). **d** O K-edge of the AS-MoS_2_ film. **e** Comparison between the O K-edges of the AS-MoS_2_ film (yellow line) and MoS_2_/SiO_2_ interface (red line). **f** Region of TR-MoS_2_/SiO_2_ in which EELS spectra were obtained. **g** S L-edge of the TR-MoS_2_ film **h** Comparison between the S L-edges of the TR-MoS_2_ film (blue line) and MoS_2_/SiO_2_ interface (red line). i O K-edge of the TR-MoS_2_ film. **j** Comparison between the O K-edges of the TR-MoS_2_ film (yellow line) and MoS_2_/SiO_2_ interface (red line)

MoS_2_ films were analyzed using X-ray photoelectron spectroscopy (XPS) to determine the chemical composition and atomic ratios of the films on the SiO_2_/Si substrate. The core level spectra of Mo 3d and S 2p were recorded for the AS- and TR-MoS_2_ films (Additional file 1: Figure S4a–d). Figure 4 shows that the AS- and TR-MoS_2_ films were both deposited in a stoichiometric composition (Mo:S = 1:2) without any significant chemical shift. In addition, the XPS characteristics of the AS-MoS_2_ film were not significantly different from those of the transferred sample. However, since the detection depth of XPS was only a few nanometers and XPS detected the overall area of the MoS_2_ films, the XPS profiles of the MoS_2_ films could not clarify the electronic structure of the MoS_2_/SiO_2_ interfaces, which are defined in the sub-nanometer range.

To identify the chemical bonding state at the MoS_2_/SiO_2_ interface, we obtained and analyzed the EELS SL and O K-edge spectra. Unlike XPS, EELS can provide chemical information in a local area with a spatial resolution in the sub-nanometer range, thus facilitating the analysis of the MoS_2_/SiO_2_ heterointerfaces. The TEM sample was thin enough for the noise in the EELS spectra to be minimized, and each spectrum was acquired at a distance of 0.6 nm. Figure 4(a) and (e) show the STEM HAADF image of both samples from which the EELS spectra were acquired; both films were found to be well attached to the substrate through the TEM sample preparation. Next, we compared the S L- and O K-edge spectra in the two above-mentioned samples to identify the bonding state of AS-MoS_2_ on the SiO_2_/Si substrate. Figure 4(b) shows the changes in the S L-edge at 10 different positions, and Fig. 4(c) shows the difference in the S L-edge spectra at the MoS_2_/SiO_2_ interface (red solid line) and the MoS_2_ film (blue solid line). The two spectra showed differences not only in the intensity, but also in the overall edge structure. In addition, considering that the MoS_2_/SiO_2_ interface was S–O-terminated, the difference of the S L-edge at the interface.Please provide complete details for the References The appearance of the peak at 168 eV indicates that the sulfur atoms were partially bonded to the oxygen atoms. The deviation from the previous study is due to the defects at the AS-MoS_2_/SiO_2_ interface. In other words, at the MoS_2_/SiO_2_ interface, the sulfur atom of the MoS_2_ film had four nearest-neighbor oxygen atoms from the amorphous SiO_2_ growth template. Figure 4(d) shows the O K-edge at 10 different positions. The O K-edge also showed a peak shift at the AS-MoS_2_/SiO_2_ interface compared to that of the interior of SiO_2_. Figure 4(c) shows that along with the S–O bond, several defects were also formed at the AS-MoS_2_/SiO_2_ interface over the range of 1.2–1.5 nm. In addition, the negative peak shifts of the SL and O K-edges at the AS-MoS_2_/SiO_2_ interface were due to an increase in the negative charge around the sulfur and oxygen ions.

The EELS edge spectra of the TR-MoS_2_/SiO_2_/Si heterostructure were also observed. Figure 4(g) and (i) present the S L- and O K-edges at 10 different positions. In contrast to those for the AS-MoS_2_ sample, the sulfur and oxygen edge spectra exhibited no significant difference, as shown in Fig. 3(h) and (j). Since the TR-MoS_2_ film was detached and transferred onto the SiO_2_/Si substrate, only van der Waals interactions existed between the MoS_2_ and SiO_2_ template.

**Fig. 5:**
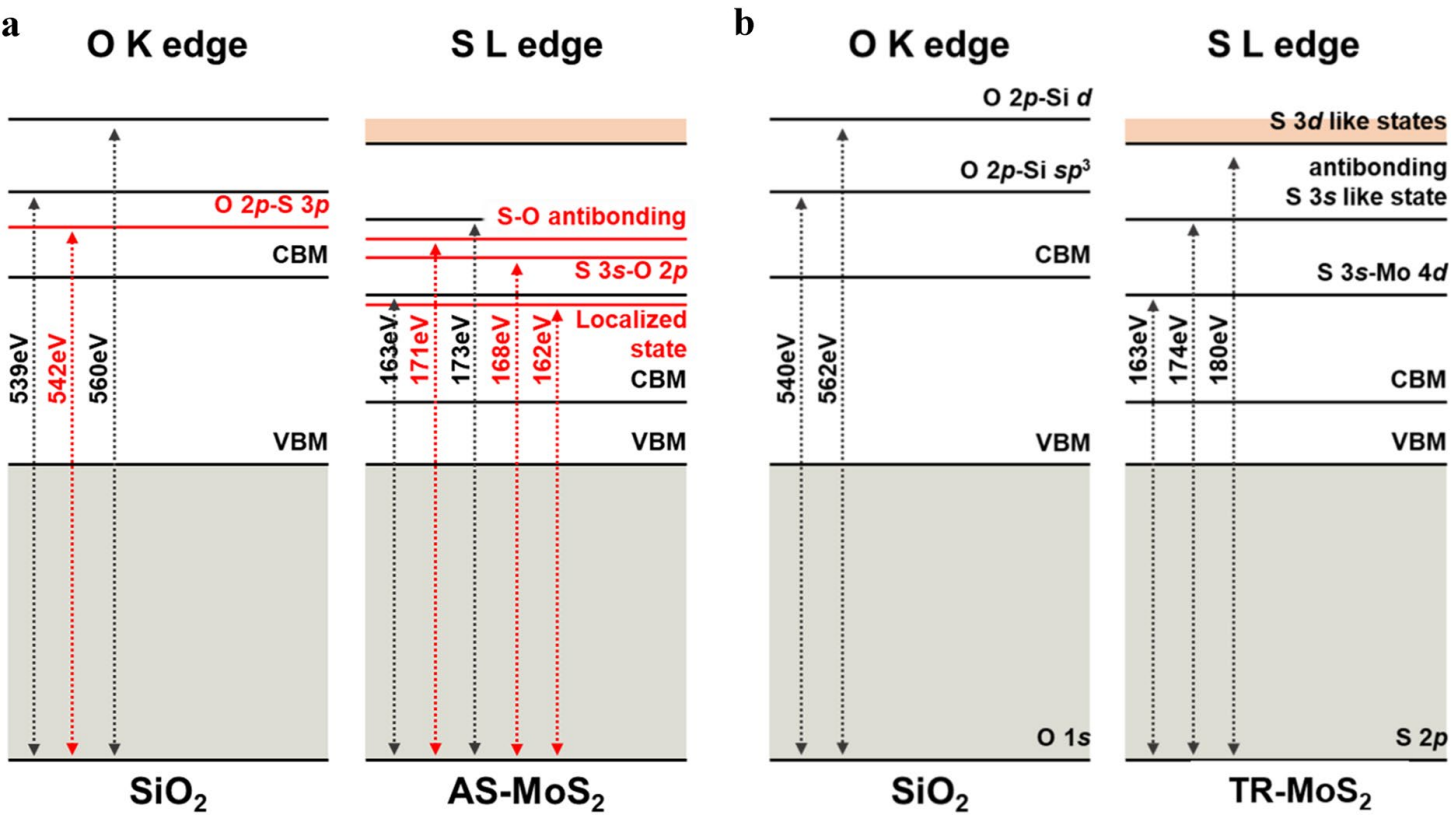
Electron transition diagram of the SiO_2_ layer **a** at the AS-MoS_2_/SiO_2_ and **b** TR-MoS_2_/SiO_2_ interface

Based on the EELS spectra of the AS-MoS_2_ sample, we prepared an electron transition diagram (Fig. 5). The EELS core loss spectra showed the transition of electrons from the core state to the unoccupied state [30]. As shown in Fig. 5(a) and (b), the peak positions near 540 eV and 560 eV in the O K-edge spectra are attributed to the transition of electrons from O 1 s to O 2p mixed with Si 3sp and Si d states [30]. Figure 5(a) shows negative peak shifts at the interface and the formation of an energy loss peak at 542 eV, which were due to an increase in the negative charge at the interface and the formation of the S–O bond, respectively. However, there is no peak shift in O K edge at the TR-MoS_2_/SiO_2_ interface, as shown in Fig. 5(b). This indicates that when sulfur atoms are chemically bonded with oxygen in SiO_2_, excess negative charges accumulate at the interface, causing a negative peak shift of the S L- and O K-edges.

**Fig. 6:**
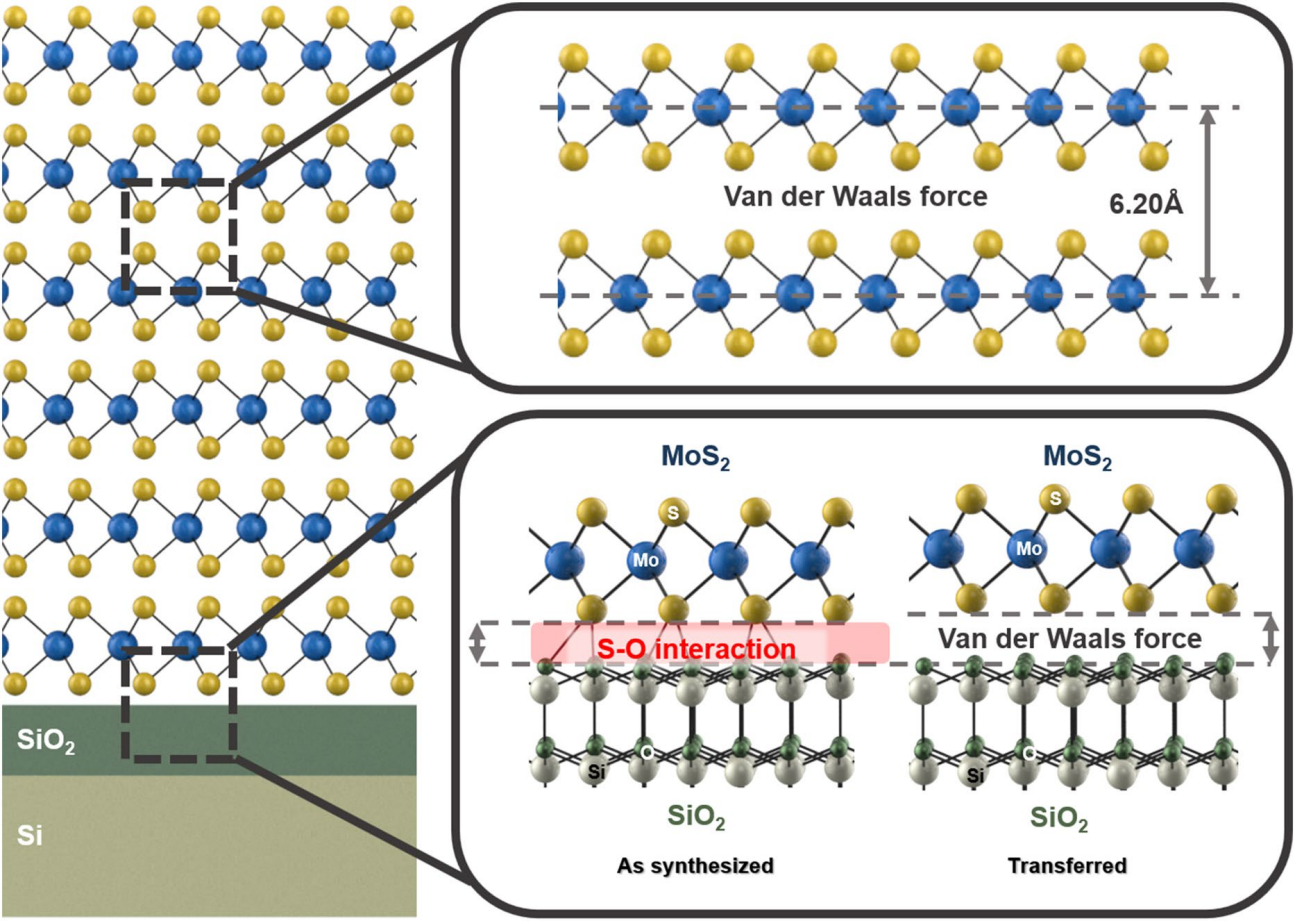
Schematic illustration of the phenomena at the AS and TR-MoS_2_/SiO_2_ interface. At the AS-MoS_2_/SiO_2_ interface, the interlayer distance decreases due to the formation of S–O bonding, whereas there is no significant change in the interlayer distance at the TR-MoS_2_/SiO_2_ interface

Combining all experimental results, the summary of our study is illustrated in Fig. 6. At the AS-MoS_2_/SiO_2_ interface, the interlayer distance decreases due to the formation of S–O bonding, whereas there is no significant change in the interlayer distance at the TR-MoS_2_/SiO_2_ interface, 12 which can explain the role of oxide templates such as SiO_2_ and Al_2_O_3_ on the large-scale growth of the MoS_2_ film. In addition, our key findings play a role in enhancing the carrier mobility of the MoS_2_ film, which can lead to the improved performance of devices [38–43].

## Conclusions

We prepared MoS_2_ films on SiO_2_/Si substrates and studied the effect of the amorphous SiO_2_ layer on the atomic and electronic structure of the MoS_2_ films. The interlayer distance of the AS-MoS_2_ film exhibited a change at the AS-MoS_2_/SiO_2_ interface, which was attributed to the formation of S–O chemical bonding at the interface. Through theoretical calculations, we confirmed the existence of a bonding state in addition to the van der Waals force, which was the dominant interaction between MoS_2_ and SiO_2_. The formation of S–O bonding at the AS-MoS_2_/SiO_2_ interface layer suggested that during CVD, the Mo thin film was not only sulfurized, but the sulfur atoms at the termination layer were also bonded to the oxygen atoms of the SiO_2_ layer, preventing the formation of Si-S bonding and MoSi_2_ (Fig. 6). Our key findings in the study are consistent regardless of the deposition techniques. In other words, the formation of S–O bonding occurs and interlayer distance between the AS-MoS_2_ film and the substrate is affected by the SiO_2_ growth template even if MoS_2_ is deposited by MOCVD or other deposition techniques.This study not only provides a guideline on the relationship between the interfacial structure and electrical properties of MoS_2_ thin film-based heterostructures and explains the role of oxides on the growth of MoS_2_ films, but also shows that this kind of interfacial interaction is prominent when it comes to single layer MoS_2_ which is generally used for a wide variety of devices.

## Supplementary Information

**Additional file 1**: **Figure S1**. Raman spectra of the MoS2 thin film on the SiO2/Si substrate. Lateral growth of multilayer MoS2 film has been successful. The two characteristic Raman vibration modes E12g and A1g are labelled. **Figure S2**. TEM images of MoS2 film on SiO2/Si. (a) Low magnification, (b), (c) HRTEM images of AS-MoS2 film. (d) Low magnification, (e), (f) HRTEM images of TR-MoS2 film. **Figure S3**. (a) Position in which interlayer distance values are measured in AS-MoS2 films and (b) position in which interlayer distance values are measured in TR-MoS2 films. **Figure S4**. XPS spectra of MoS2 films. XPS core level spectra of (a) Mo 3d, (b) S 2p of AS-MoS2 film and (c) Mo 3d, (d) S 2p of TR-MoS2 films.

## Data Availability

Not applicable.

## Abbreviations

AS-MoS_2_: As-synthesized MoS_2_
TR-MoS_2_: Transferred MoS_2_
STEM: Scanning transition electron microscopy
EELS: Electron energy loss spectroscopy
CVD: Chemical vapor deposition
PLD: Pulsed laser deposition
TEM: Transmission electron microscopy
Cs-corrected STEM: Aberration-corrected scanning transmission electron microscopy
HAADF: High annular dark field
ABF: Annular bright field
DI: Deionized
VASP: *Vienna *Ab initio Simulation package
DFT: Density functional theory
GGA: Generalized gradient approximation

## References

[1] Liu X, Chen L, Liu Q, He J, Li K, Yu W, Ao JP, Ang KW (2017). J. Alloys Compd..

[2] Novoselov KS, Jiang D, Schedin F, Booth TJ, Khotkevich VV, Morozov SV, Geim AK (2005). Proc. Natl. Acad. Sci. U. S. A..

[3] Radisavljevic B, Radenovic A, Brivio J, Giacometti V, Kis A (2011). Nat. Nanotechnol..

[4] Kwon KC, Choi S, Hong K, Moon CW, Shim YS, Kim DH, Kim T, Sohn W, Jeon JM, Lee CH, Nam KT, Han S, Kim SY, Jang HW (2016). Energy Environ. Sci..

[5] Laskar MR, Ma L, Kannappan S, Sung Park P, Krishnamoorthy S, Nath DN, Lu W, Wu Y, Rajan S (2013). Appl. Phys. Lett..

[6] Coleman JN, Lotya M, O’Neill A, Bergin SD, King PJ, Khan U, Young K, Gaucher A, De S, Smith RJ, Shvets IV, Arora SK, Stanton G, Kim HY, Lee K, Kim GT, Duesberg GS, Hallam T, Boland JJ, Wang JJ, Donegan JF, Grunlan JC, Moriarty G, Shmeliov A, Nicholls RJ, Perkins JM, Grieveson EM, Theuwissen K, McComb DW, Nellist PD, Nicolosi V (2011). Science.

[7] Lee MWSWY, Besmann TM (1994). J. Mater. Res..

[8] Lee YH, Zhang XQ, Zhang W, Chang MT, Te Lin C, Di Chang K, Yu YC, Wang JTW, Chang CS, Li LJ, Lin TW (2012). Adv. Mater..

[9] Clark G, Wu S, Rivera P, Finney J, Nguyen P, Cobden DH, Xu X (2014). APL Mater..

[10] Ye M, Winslow D, Zhang D, Pandey R, Yap YK (2015). Photonics.

[11] Lu N, Zhang C, Lee CH, Oviedo JP, Nguyen MAT, Peng X, Wallace RM, Mallouk TE, Robinson JA, Wang J, Cho K, Kim MJ (2016). J. Phys. Chem. C.

[12] Deokar G, Rajput NS, Vancsó P, Ravaux F, Jouiad M, Vignaud D, Cecchet F, Colomer JF (2017). Nanoscale.

[13] Nguyen TP, Sohn W, Oh JH, Jang HW, Kim SY (2016). J. Phys. Chem. C.

[14] Su X, Cui H, Ju W, Yong Y, Li X (2017). Mod. Phys. Lett. B.

[15] Bertrand PA (1989). Langmuir.

[16] Hussain S, Singh J, Vikraman D, Singh AK, Iqbal MZ, Khan MF, Kumar P, Choi DC, Song W, An KS, Eom J, Lee WG, Jung J (2016). Sci. Rep..

[17] Zhou W, Zou X, Najmaei S, Liu Z, Shi Y, Kong J, Lou J, Ajayan PM, Yakobson BI, Idrobo JC (2013). Nano Lett..

[18] Wu RJ, Odlyzko ML, Mkhoyan KA (2014). Ultramicroscopy.

[19] Jin L, Jia CL, Lindfors-Vrejoiu I, Zhong X, Du H, Dunin-Borkowski RE (2016). Adv. Mater. Interfaces.

[20] Ishikawa Y, Wada K, Cannon DD, Liu J, Luan HC, Kimerling LC (2003). Appl. Phys. Lett..

[21] Siow KS, Britcher L, Kumar S, Griesser HJ (2018). Sains Malaysiana.

[22] Cho DY, Tappertzhofen S, Waser R, Valov I (2013). Nanoscale.

[23] Kresse G, Furthmüller J (1996). Comput. Mater. Sci..

[24] Kresse JFG (1996). Phys. Rev. B.

[25] Hohenberg P, Kohn W (1964). Phys. Rev..

[26] Kohn W, Sham LJ (1965). Phys. Rev..

[27] Li H, Zhang Q, Yap CCR, Tay BK, Edwin THT, Olivier A, Baillargeat D (2012). Adv. Funct. Mater..

[28] Yu Y, Li C, Liu Y, Su L, Zhang Y, Cao L (2013). Sci. Rep..

[29] Najmaei S, Liu Z, Zhou W, Zou X, Shi G, Lei S, Yakobson BI, Idrobo JC, Ajayan PM, Lou J (2013). Nat. Mater..

[30] Sung JH, Heo H, Si S, Kim YH, Noh HR, Song K, Kim J, Lee CS, Seo SY, Kim DH, Kim HK, Yeom HW, Kim TH, Choi SY, Kim JS, Jo MH (2017). Nat. Nanotechnol..

[31] Tan LK, Liu B, Teng JH, Guo S, Low HY, Loh KP (2014). Nanoscale.

[32] Tan CS, Lu YJ, Chen CC, Liu PH, Gwo S, Guo GY, Chen LJ (2016). J. Phys. Chem. C.

[33] R.H. and Y.I.X. Gao, Y.H. Ikuhara, C.A. Fisher, H. Moriwake, A. Kuwabara, H. Oki, K. Kohama, R. Yoshida, Adv. Mater. Interfaces **1**, 1400143 (2014)

[34] Petrov PK, Zou B, Wang Y, Perkins JM, McComb DW, Alford NMN (2014). Adv. Mater. Interfaces.

[35] Blöchl PE (1994). Phys. Rev. B.

[36] Kresse G, Joubert D (1999). Phys. Rev. B Condens. Matter Mater. Phys..

[37] Perdew JP, Burke K, Ernzerhof M (1996). Phys. Rev. Lett..

[38] Yu Z, Ong ZY, Li S, Xu JB, Zhang G, Zhang YW, Shi Y, Wang X (2017). Adv. Funct. Mater..

[39] Suh JM, Shim Y-S, Kwon KC, Jeon J-M, Lee TH, Shokouhimehr M, Jang HW (2019). Electron. Mater. Lett..

[40] Wang B, Muratore C, Voevodin AA, Haque MA (2014). Nano Converg..

[41] Andoshe DM, Jeon J-M, Kim SY, Jang HW (2015). Electron. Mater. Lett..

[42] Shin HW, Son JY (2018). Electron. Mater. Lett..

[43] Zhang F-J, Kong C, Li X, Sun X-Y, Xie W-J, Oh W-C (2019). J. Korean. Ceram. Soc.

