# Supplementary material for: Microscopic evidence of strong interactions between chemical vapor deposited 2D MoS2 film and SiO2 growth template
Source: Nano Converg. 2021 Apr 9;8:11. doi: 10.1186/s40580-021-00262-x (PMC8032840; doi:10.1186/s40580-021-00262-x)
Supplement: Supplementary file 1 — Additional file 1: Figure S1. Raman spectra of the MoS2 thin film on the SiO2/Si substrate. Lateral growth of multilayer MoS2 film has been successful. The two characteristic Raman vibration modes E12g and A1g are labelled. Figure S2. TEM images of MoS2 film on SiO2/Si. (a) Low magnification, (b), (c) HRTEM images of AS-MoS2 film. (d) Low magnification, (e), (f) HRTEM images of TR-MoS2 film. Figure S3. (a) Position in which interlayer distance values are measured in AS-MoS2 films and (b) position in which interlayer distance values are measured in TR-MoS2 films. Figure S4. XPS spectra of MoS2 films. XPS core level spectra of (a) Mo 3d, (b) S 2p of AS-MoS2 film and (c) Mo 3d, (d) S 2p of TR-MoS2 films. [file 40580_2021_262_MOESM1_ESM.docx]

**Supplementary Information**

**Microscopic evidence of strong interactions between chemical vapor deposited 2D MoS_2_ film and SiO_2_ growth template**

Woonbae Sohn^1, 2^, Ki Chang Kwon^1^, Jun Min Suh^1^, Tae Hyung Lee^1^, Kwang Chul Roh^*2^ and Ho Won Jang^*1^

*^1)^ Department of Materials Science and Engineering, Research Institute of Advanced Materials, Seoul National University, Seoul 08826, Republic of Korea.*

*^2)^ Energy Storage Materials Centre, Korea Institute of Ceramic Engineering and Technology, Jinju 52851, Republic of Korea.*

*E-mail address: hwjang@snu.ac.kr, rkc@kicet.re.kr, Tel: 82-2-880-1720*

**
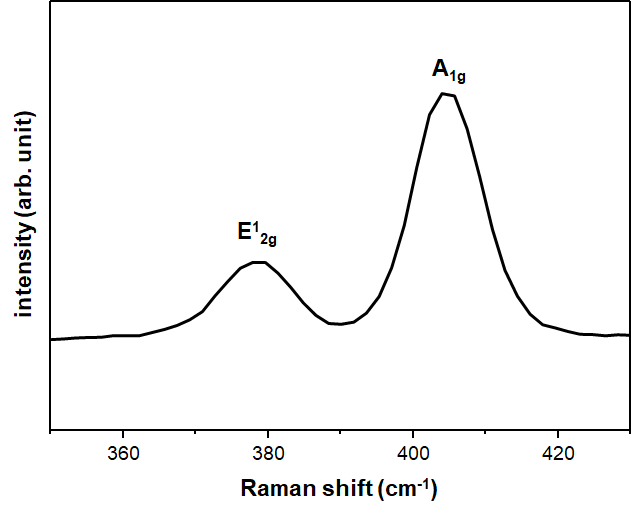
**

**Figure S1.** Raman spectra of the MoS_2_ thin film on the SiO_2_/Si substrate. Lateral growth of multilayer MoS_2_ film has been successful. The two characteristic Raman vibration modes E^1^_2g_ and A_1g_ are labelled


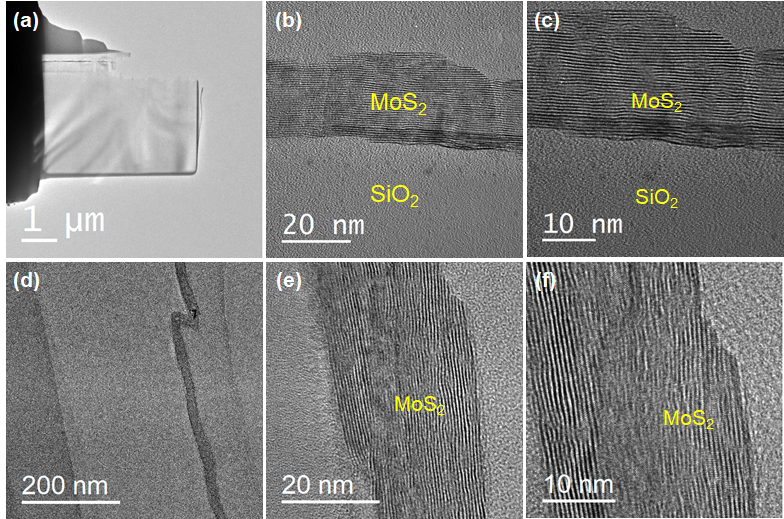


**Figure S2**. TEM images of MoS_2_ film on SiO_2_/Si. (a) Low magnification, (b), (c) HRTEM images of AS-MoS_2_ film. (d) Low magnification, (e), (f) HRTEM images of TR-MoS_2_ film.


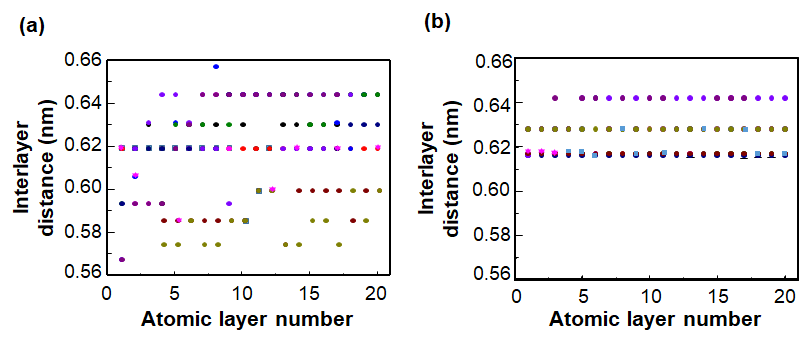


**Figure S3.** (a) Position in which interlayer distance values are measured in AS-MoS_2_ films and (b) position in which interlayer distance values are measured in TR-MoS_2_ films.

**
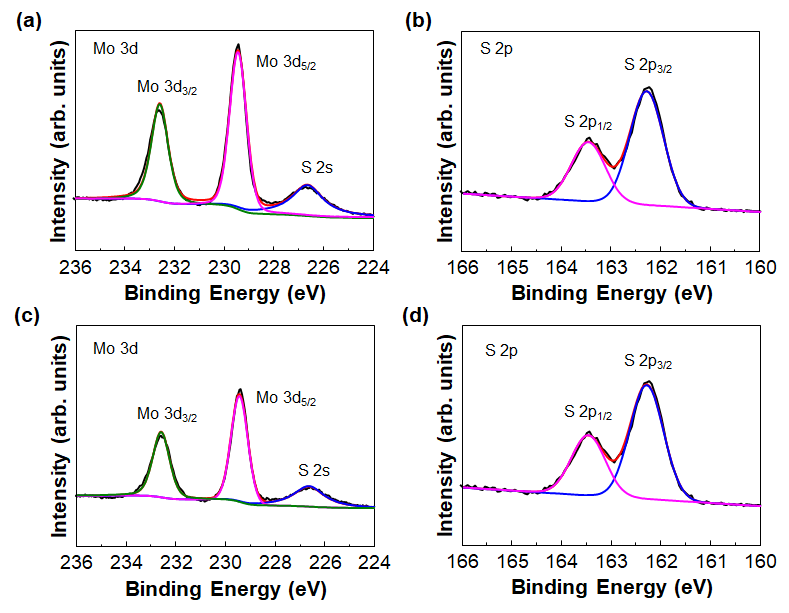
**

**Figure S4.** XPS spectra of MoS_2_ films. XPS core level spectra of (a) Mo 3d, (b) S 2p of AS-MoS_2_ film and (c) Mo 3d, (d) S 2p of TR-MoS_2_ films.
